# *MET* amplification identified by next-generation sequencing and its clinical relevance for* MET* inhibitors

**DOI:** 10.1186/s40164-021-00245-y

**Published:** 2021-11-10

**Authors:** Lun-Xi Peng, Guang-Ling Jie, An-Na Li, Si-Yang Liu, Hao Sun, Mei-Mei Zheng, Jia-Ying Zhou, Jia-Tao Zhang, Xu-Chao Zhang, Qing Zhou, Wen-Zhao Zhong, Jin-Ji Yang, Hai-Yan Tu, Jian Su, Hong-Hong Yan, Yi-Long Wu

**Affiliations:** 1 The Second School of Clinical Medicine, Southern Medical University, Guangzhou, China; 2 Guangdong Lung Cancer Institute, Guangdong Provincial Key Laboratory of Translational Medicine in Lung Cancer, Guangdong Provincial People’s Hospital & Guangdong Academy of Medical Sciences, Guangzhou, 510080 China; 3 School of Medicine, South China University of Technology, Guangzhou, China

**Keywords:** FISH-NGS, *MET* amplification, Predictive factors, Survival benefits

## Abstract

**Background:**

*MET* amplification plays an important role in the development of non-small-cell lung cancer (NSCLC) either de novo or in resistance to epidermal growth factor receptor tyrosine–kinase inhibitor (EGFR-TKI) settings. Fluorescence in situ hybridization (FISH) is the standard method for *MET* amplification. With more and more discoveries of oncogenic driver genes, next-generation sequencing (NGS) plays a significant role in precision oncology. Meanwhile, the role of NGS in *MET* amplification remains uncertain.

**Methods:**

Forty patients diagnosed with advanced NSCLC were included. FISH and NGS were conducted prior to MET inhibitors treatment. *MET* amplification by FISH was defined as a MET/CEP7 ratio of  >  2.0 and/or copy number (CN)  >  5. *MET* amplification by NGS was defined as gene copy number (GCN)  ≥  5.

**Results:**

The concordance rate among FISH and NGS was 62.5% (25/40). *MET* amplification identified by FISH showed the optimal predictive value. The partial response (PR) rate was 68.0% (17/25 with *MET* amplification) vs. 6.7% (1/15 without *MET* amplification); the median progression-free survival (PFS) was 5.4 months versus 1.0 months (P  < 0.001). *MET* amplification identified by NGS failed to distinguish significant clinical outcomes. The PR rate was 60.0% (6/10, with *MET* GCN  ≥ 5) vs. 40.0% (12/30, with *MET* GCN  < 5); the median PFS was 4.8 months vs. 2.2 months (P  = 0.357). The PR rate was 68.8% (11/16) and the median PFS was 4.8 months in patients with focal amplification by NGS.

**Conclusions:**

*MET* amplification identified by FISH remains the optimal biomarker to identify suitable candidates for MET-TKI therapy. In comparison, amplification identified by NGS seems not as robust to be effective predictive biomarker. Further exploration is needed regarding the focal amplification by NGS in predicting the efficacy.

**Supplementary Information:**

The online version contains supplementary material available at 10.1186/s40164-021-00245-y.

## Background

The discovery of oncogenic driver genes has improved the overall survival (OS) of advanced non-small-cell lung cancer (NSCLC) patients in clinical practice [1–4]. One of the most important achievements in NSCLC research has been the development of epidermal growth factor receptor tyrosine kinase inhibitors (EGFR-TKIs), which have increased the OS of patients with advanced-stage EGFR-mutated NSCLC to approximately 22–34 months [5–9]. Small molecular drugs targeting eight oncogenic driver genes (*EGFR*, *ALK*, *ROS1*, *BRAF* V600E, *MET* exon 14 skipping, *RET*, *KRAS* G12C, and *NTRK*) have been approved for the market [10]. The search for new targets has become an important direction in lung cancer research.

*MET*, located in the 7q21-31 region, belongs to the tyrosine kinase protein family. *MET* activation has been shown to promote tumor cell growth, survival, migration, and invasion by interacting with multiple pathways [11]. Major types of *MET* abnormalities include the *MET* exon 14 skipping mutation, *MET* amplification, MET overexpression, and *MET* fusion. The *MET* exon 14 skipping mutation exists in approximately 5% of lung cancer patients and has been recognized as an oncogenic driver gene [12]. MET overexpression is common in untreated NSCLC patients, occurring in approximately 50% of this population. De novo *MET* amplification occurring in only 1–5% of NSCLC patients. However, among patients who develop EGFR-TKI resistance, 64% and 5–22% of patients show MET overexpression and *MET* amplification, respectively [13, 14].

*MET* amplification represents a resistance mechanism in *EGFR-*mutated NSCLC patients treated with EGFR-TKIs [15–18]. The INC280 study showed that in patients with *EGFR* mutation and acquired *MET* amplification, the combination of capmatinib with gefitinib was a promising treatment, with a disease control rate of 57%. Notably, the objective response rate (ORR) was up to 47% in patients with *MET* amplification, defined as Copy number (CN)  ≥ 6 [19]. Tepotinib plus gefitinib also showed significantly better progression-free survival (PFS) and OS than chemotherapy in patients with *MET* amplification (16.6 months vs. 4.2 months; 37.3 months vs. 17.9 months, respectively) [20]. Two phase Ib clinical trials revealed that a combination of savolitinib and osimertinib or gefitinib showed promising antitumor activity and tolerable toxicity in patients with acquired *MET*-amplified NSCLC [21, 22].

Moreover, *MET* amplification, particularly at a high level, also seems to play a driver gene role in advanced NSCLC. A previous study showed that the ORR in groups with different MET/CEP7 ratios differed dramatically in response to the *MET*/*ALK*/*ROS1* inhibitor crizotinib (MET/CEP7  ≥ 5, ORR  = 67%) [23]. A recent study also founded better survival benefits in patients with MET/CEP7  ≥ 4 (ORR of 38.1%, median PFS  = 6.7 months) [24]. Presentations during the 2020 American Society of Clinical Oncology and 2020 European Society of Medical Oncology meetings reported that capmatinib had achieved higher ORR (40.0%) and better survival benefits in patients with de novo* MET* amplification, especially with high-level *MET* amplification [gene copy number (GCN)  ≥ 10] as a first-line treatment [25, 26].

With respect to diagnostic modalities for *MET* amplification, fluorescence in situ hybridization (FISH) was considered the gold standard. A previous study showed that PFS differed significantly between patients with *MET* amplification-positive and -negative FISH findings (8.2 months vs. 1.3 months, P  = 0.002) [27]. Recently, next-generation sequencing (NGS) has been widely applied in clinical practice to detect comprehensive gene profiles, including point testing of multiple-gene DNA mutations, as well as gene amplification, rearrangement, and fusion. However, the definition of *MET* amplification varies on different NGS platforms. The cutoff value varies from GCN 2.3–10. It remains unclear whether NGS can serve as an alternate method for identifying *MET* amplification. Therefore, we conducted this study to investigate the relationship between *MET* amplification detected by FISH and *MET* amplification detected by NGS. We then explored optimal biomarkers based on their efficacy in selection of suitable candidates for MET-TKI treatment in advanced NSCLC.

## Patients and methods

This study was conducted at the Guangdong Lung Cancer Institute and was approved by the Research Ethics Committee of the Guangdong Provincial People’s Hospital (No. 2013185H[R2]). Written informed consent was obtained from each patient prior to sample collection. From March 2014 to June 2019, 40 NSCLC patients with *MET* inhibitors were included in this study. Immunohistochemistry (IHC) was conducted for primary screening and then *MET* amplification was tested by FISH and NGS at baseline for all patients. Tumor samples were tested using FISH and NGS to identify *MET* amplification prior to MET-TKI. Baseline clinicopathological data, including patient characteristics and gene status (*EGFR* and *MET*), were collected from medical records. PFS was measured from the date of first administration of MET-TKIs until the date of disease progression or death. Response rate and PFS were calculated separately for FISH and NGS results, then used to compare the two methods of testing.

### Assessment of *MET* amplification

#### Fluorescence in situ hybridization

Dual-color FISH was performed using deparaffinized 4-μm-thick sections with a MET/CEN7q dual-color FISH probe (Vysis, Abbott Laboratories). *MET* amplification was defined as a mean gene copy number  ≥ 5 and/or MET to centromere of chromosome 7 ratio  > 2.0 and evaluated using the criteria established by Cappuzzo [22] (i.e., a mean of  > 5 copies per cell, MET-to-CEN7 ratio of  > 2.0 or clustered gene amplification evident in all nuclei).

#### Next-generation sequencing

NGS was performed using a HiSeq 4000 NGS platform (Illumina) or NovaSeq 6000 NGS platform (Illumina). *MET* amplification was evaluated based on the ratio of GCN to a baseline established from a pool of samples with normal MET status. GCN  ≥ 5 was defined as *MET* amplification criteria from TATTON trial [22]. The criteria were samples with  ≥ 10% tumor cells and  ≥ 500 ×  sequencing depth. Further analysis was conducted to distinguish between focal and non-focal *MET* amplification, where focal amplification was defined as a *MET* amplification size of  < 20 Mbp or both *MET*/*CDK6* and *MET*/*BRAF* ratios of  ≥ 1.2.

## Results

**Fig. 1:**
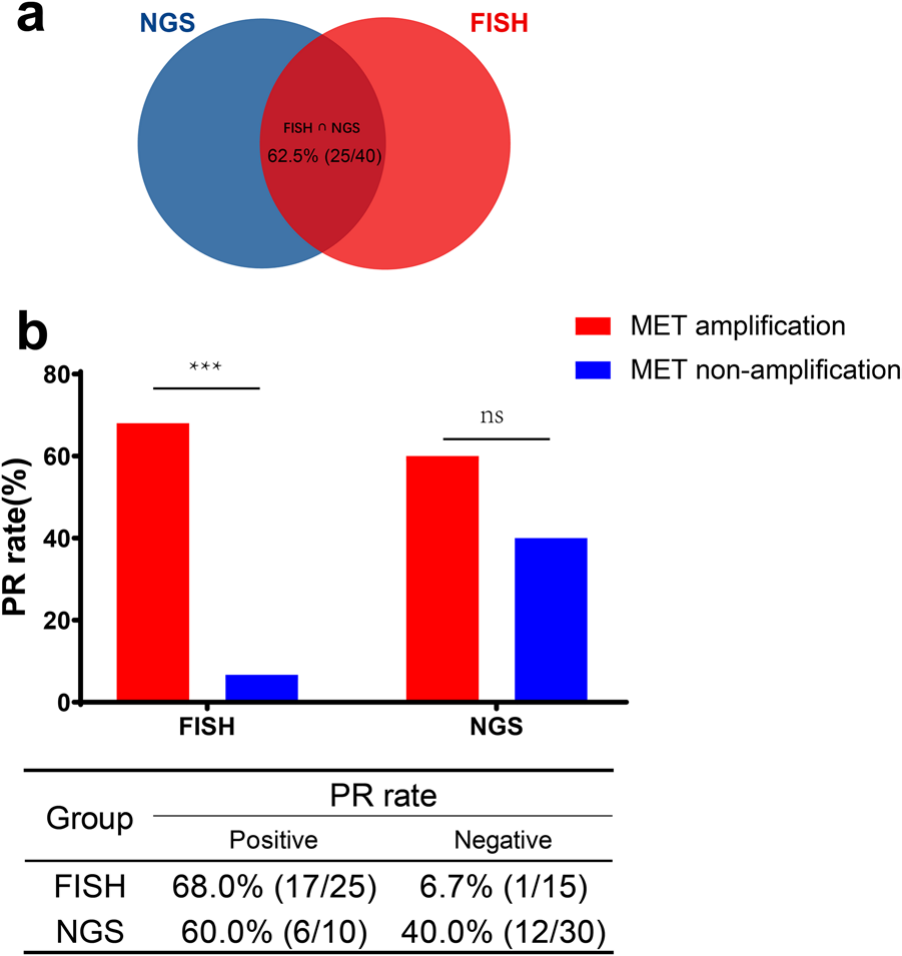
Comparison between MET amplification identified by FISH and NGS. **a** Consistency between *MET* amplification identified by FISH and NGS. **b** PR rate in group between *MET* amplification and MET non-amplification identified by different methods. ***P value  < 0.001, *ns* no significance

**Fig. 2:**
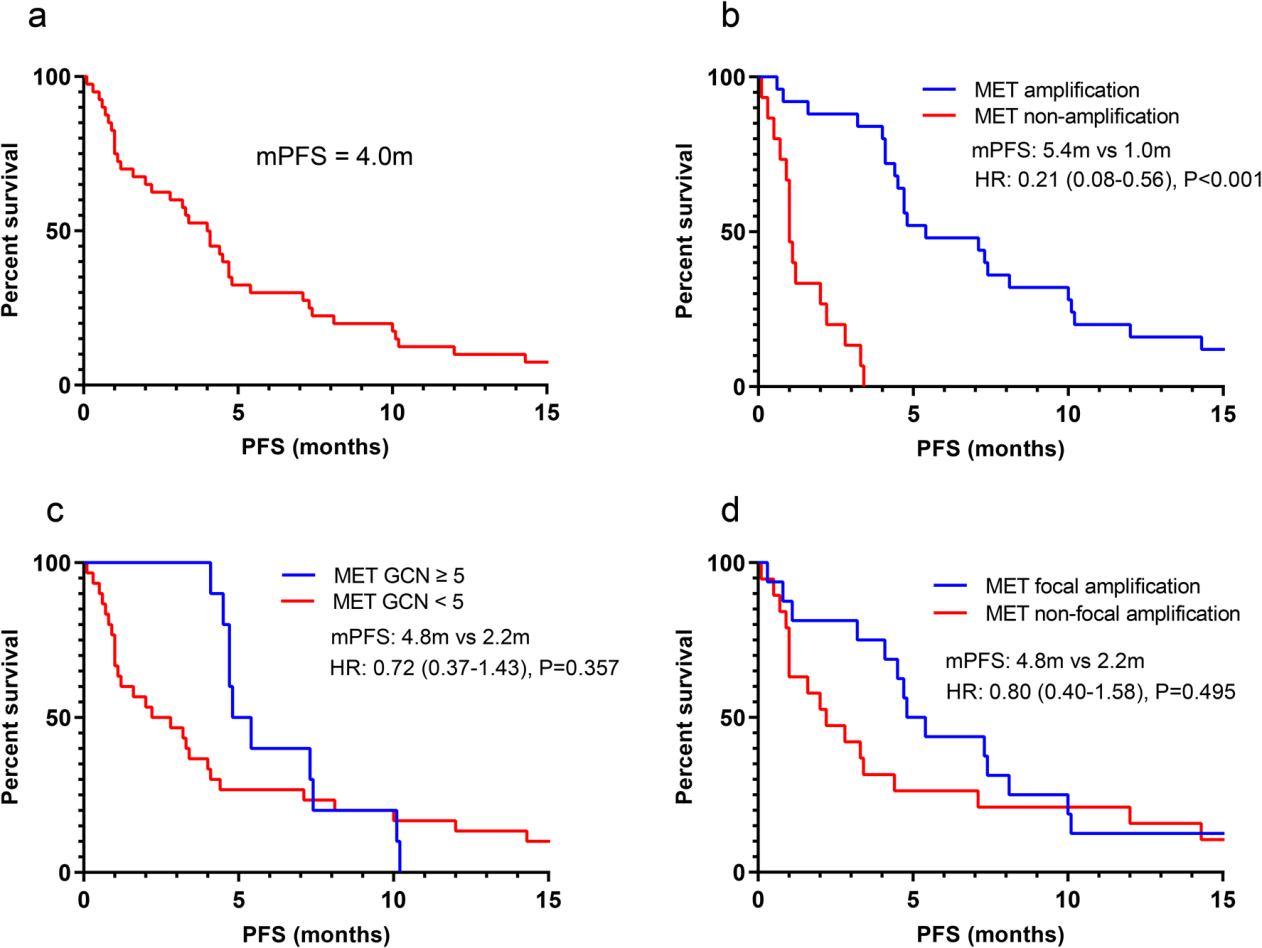
Progression-free survival in patients. **a** Progression-free survival in all patients (n  =  40). **b** Progression-free survival between *MET* amplification/non-amplification by FISH (n = 40). **c** Progression-free survival between *MET* GCN ≥  5/GCN <  5 by NGS (n = 40). **d** Progression-free survival between *MET* focal amplification/non-focal amplification by NGS (n  = 35)

From March 2014 to June 2019, 40 NSCLC patients with *MET* inhibitors were included in this study. IHC was conducted for primary screening and then *MET* amplification was tested by FISH and NGS at baseline for all patients (Table 1). Among all tests, 25/40 cases of *MET* amplification were diagnosed by FISH and 10/40 cases were diagnosed by NGS (GCN  ≥ 5). The concordance rate between FISH and NGS was 62.5% (25/40) (concordance rate means the same results from FISH and NGS, including negative or positive) (Fig. 1a). All patients received *MET* inhibitors (such as crizotinib, Savolitinib and bozitinib etc.) as treatment. The partial response (PR) rate was 45.0% (18/40) and the median PFS was 4.0 months (Fig. 2a).

**Fig. 3:**
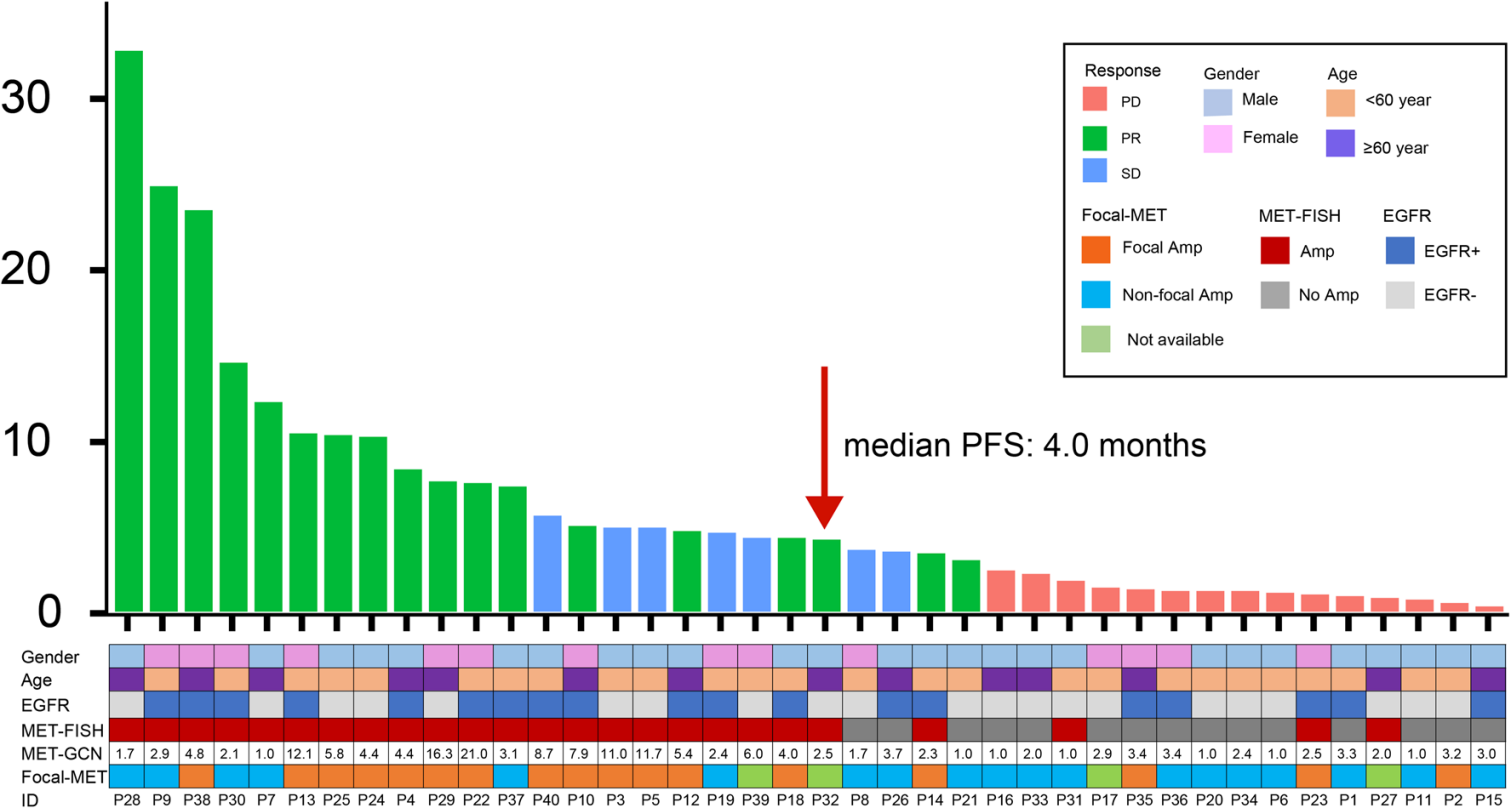
Explorations for GCN and survival. Amp: MET amplification identified by FISH; No Amp: non-*MET* amplification identified by FISH; Focal Amp: *MET* focal amplification by NGS; Non-Focal Amp: *MET* non-focal amplification by NGS; *GCN* gene copy number

We calculated the PR rate and PFS for *MET* amplification identified by FISH and NGS. In the FISH group, the PR rate was 68.0% (17/25) vs. 6.7% (1/15); the median PFS was 5.4 months vs. 1.0 months (*P*  < 0.001) (Figs. 1b, 2b). In the NGS group, the PR rate was 60.0% (6/10) vs. 40.0% (12/30); the median PFS was 4.8 months vs. 2.2 months (*P*  = 0.357) (Figs. 1b, 2c). Among the 35 available tumor samples, 45.7% (16/35) were identified as *MET* focal amplification by NGS (Fig. 3). The PR rate was 68.8% (11/16) and the median PFS was 4.8 months in patients with *MET* focal amplification by NGS (Fig. 2d).

Multivariable analyses of PFS using the Cox proportional hazard regression method showed that PFS was significantly different only according to the *MET* amplification identified by FISH.

## Discussion

*MET* amplification as a pharmaceutical target has become a hot research topic, especially in terms of EGFR-TKI resistance. Unlike gene point mutation, *MET* amplification is difficult to test using reverse-transcription polymerase chain reaction (RT-PCR) or NGS. Although NGS results are widely used to guide the clinical management of oncogene driver-positive cancer, there is a lack of evidence regarding the accuracy and appropriateness of *MET* amplification identified by NGS as an indication for MET-TKI treatment. Few studies have investigated the concordance between NGS and FISH detection results for *MET* amplification. One study reported a low correlation of *MET* amplification results obtained by NGS and FISH. Among patients with FISH-positive results who had CN  ≥ 8, only 1/3 exhibited *MET* amplification according to NGS [28]. The results of the TATTON study also showed low consistency between NGS and FISH for *MET* amplification. In the FISH-positive group, only 26% of the patients (12/47) were diagnosed with *MET* amplification by NGS [29, 30].

In our study, *MET* amplification identified by FISH showed the most optimal predictive efficiency for survival benefits. The PR rate [68.0% (17/25) vs. 6.7% (1/15)] and the median PFS (5.4 months vs. 1.0 months) were higher in the *MET* amplification group than in the non-*MET* amplification group (*P*  < 0.001). Our findings showed that FISH positivity remains as the “gold standard” for evaluating *MET* amplification, with high accuracy and good correlation with treatment outcomes. Several patients such as ID23, got progression although they were carrying *MET-*amplification by FISH. It was indicating that some co-occurring gene alterations have potential to affect response, including *TP53* mutation, *EGFR* amplification.

*MET* amplification identified by NGS failed to distinguish significant clinical outcomes. In the NGS group, the PR rate of *MET* amplification and non-amplification was 60.0% (6/10) vs. 40.0% (12/30); the median PFS was 4.8 months vs. 2.2 months (*P*  = 0.357) (Fig. 2c). Moreover, setting GCN  = 5 as cutoff value for MET amplification by NGS was also likely to resulting in missing patients that with response.

Under FISH, *MET* amplification is defined as *MET* CN  > 5 or MET/CEP7  > 2.0. This criterion has been applied in several clinical trials including INSIGHT, VISION, and TATTON studies [19, 31–33]. Under NGS, some recent trials found higher gene copy number may have better predictive power. For example, the TATTON study defined GCN  > 5 as the *MET* amplification. The GEOMETRY mono-1 study also found that patients with GCN ≥ 10 tended to have a better overall response [25]. Similar results were observed in our study for patients with GCN  > 5. Though only 12.5% (5/40) of patients showed GCN  ≥ 10, the PR rate among these patients reached 60.0%, with a median PFS of 7.3 months. However, patients with lung cancer based on this criterion are rare. In our study, only 8.5% (5/59) of patients showed a preliminary GCN of  > 10 (Additional file 1: Table S1).

We found no significant associations among *MET* amplification status determined by NGS with survival benefits. There remains no general consensus regarding the protocol for detecting *MET* amplification by NGS in patients with lung cancer. No internationally accepted standard for testing *MET* amplification by NGS has been established. In this study, *MET* amplification by NGS was based on the ratio of GCN to the baseline value from a pool of samples with a known normal *MET* status, such that it is difficult to discriminate true amplification and polysomy by NGS. Therefore, *MET* amplification testing by NGS remains uncertain and it could not be directly used in clinical practice. If GCN  < 5, it is recommended to confirm *MET* status by FISH (Table 1).

**Table 1:** Clinicopathologic characteristics of enrolled patients

Characteristic	N (%)
Age, years	
< 60	26 (65.0)
≥ 60	14 (35.0)
Sex	
Female	14 (35.0)
Male	26 (65.0)
Pathology	
Adenocarcinoma	37 (92.5)
Others	3 (7.5)
EGFR status	
Wide-type	21 (52.5)
Mutation	19 (47.5)
MET exon 14 skipping mutations	
Yes	2 (5.0)
No	38 (95.0)
MET amplification by FISH	
Amplified	25 (62.5)
Non-amplified	15 (37.5)
MET amplification by NGS	
GCN ≥ 5	10 (25.0)
GCN < 5	30 (75.0)
Focal amplified by NGS	
Focal amplified	16 (40.0)
Non-focal amplified	19 (47.5)
Not available	5 (12.5)

Data are presented as n (%)

*GCN* Gene copy number

Biological characteristics differ between focal and non-focal *MET* amplification diagnosed by NGS. A previous study using hybrid-capture-based comprehensive genomic profiling showed a higher median CN in patients with focal *MET* amplification than in patients with non-focal *MET* amplification (11 copies vs. 7 copies; *P * = 0.004). Furthermore, neither tumor mutation burden nor co-occurring *MET* and *EGFR* mutations were significantly correlated with the size of the *MET* amplification. However, other co-occurring oncogenic drivers were associated with non-focal *MET* amplification [34]. Therefore, we explored the role of focal *MET* amplification using NGS. The results showed a focal *MET* amplification frequency of 48.5% (17/35). The PR rate was 68.8% (11/16) and the median PFS was 4.8 months in this patient group (Fig. 2d). Furthermore, patients with focal *MET* amplification present with a significantly higher median GCN than patients with non-focal *MET* amplification (7.5 copies vs. 2.7 copies; *P*  < 0.001), suggesting that focal amplification identified by NGS and its clinical relevance warrant further research. Despite some meaningful findings obtained in this study, our small sample sizes require cautious interpretation of the results.

In conclusion, *MET* amplification identified by FISH remains the optimal biomarker to identify suitable candidates for MET-TKI therapy. In this small, exploratory series, *MET* amplification identified by NGS seems not as effective as a predictive biomarker for MET inhibitors. Further research is required regarding the role of focal amplification by NGS in predicting its efficacy.

## Supplementary Information

**Additional file 1: Table S1.** Range of GCN of total 59 patients at the preliminary.

## Data Availability

The datasets generated and/or analyzed during the current study are not publicly available but it are available from the corresponding author on reasonable request.

## Abbreviations

*EGFR*: Epidermal growth factor receptor
*ALK*: Anaplastic lymphoma kinase
*MET*: Mesenchymal epithelial transition
*RET*: RET proto-oncogene
*NTRK*: Neurotrophic receptor tyrosine kinase
*BRAF*: B-Raf proto-oncogene
EGFR-TKI: Epidermal growth factor receptor-tyrosine kinase inhibitor
FISH: Fluorescence in situ hybridization
CN: Copy number
GCN: Gene copy number
NGS: Next-generation sequencing
NSCLC: Non-small cell lung cancer
ORR: Objective response rate
OS: Overall survival
PR: Partial response
PFS: Progression-free survival
RT-PCR: Reverse-transcription polymerase chain reaction
