# Supplementary material for: MET amplification identified by next-generation sequencing and its clinical relevance for MET inhibitors
Source: Exp Hematol Oncol. 2021 Nov 10;10:52. doi: 10.1186/s40164-021-00245-y (PMC8579577; doi:10.1186/s40164-021-00245-y)
Supplement: Supplementary file 1 — Additional file 1: Table S1. Range of GCN of total 59 patients at the preliminary. [file 40164_2021_245_MOESM1_ESM.docx]

Supplementary table 1. Range of GCN of total 59 patients at the preliminary.

|  | GCN＞2.0 | GCN＞5.0 | GCN＞10.0 |
| --- | --- | --- | --- |
| N (%) | 43 (72.9%） | 12（20.3%） | 5（8.5%） |

GCN：gene copy number
